# Flow simulation-based particle swarm optimization for developing improved hemolysis models

**DOI:** 10.1007/s10237-022-01653-7

**Published:** 2022-11-28

**Authors:** B. Torner, D. Frank, S. Grundmann, F.-H. Wurm

**Affiliations:** 1 Institute of Turbomachinery, University of Rostock, Rostock, Germany; 2 Institute of Fluid Mechanics, University of Rostock, Rostock, Germany

**Keywords:** Turbulent blood flow simulation, Hemolysis modeling, Hemocompatibility, Blood trauma, Particle swarm optimization, Multi-Objective optimization, Mechanical circulatory support

## Abstract

The improvement and development of blood-contacting devices, such as mechanical circulatory support systems, is a life saving endeavor. These devices must be designed in such a way that they ensure the highest hemocompatibility. Therefore, *in-silico* trials (flow simulations) offer a quick and cost-effective way to analyze and optimize the hemocompatibility and performance of medical devices. In that regard, the prediction of blood trauma, such as hemolysis, is the key element to ensure the hemocompatibility of a device. But, despite decades of research related to numerical hemolysis models, their accuracy and reliability leaves much to be desired. This study proposes a novel optimization path, which is capable of improving existing models and aid in the development of future hemolysis models. First, flow simulations of three, turbulent blood flow test cases (capillary tube, FDA nozzle, FDA pump) were performed and hemolysis was numerically predicted by the widely-applied stress-based hemolysis models. Afterward, a multiple-objective particles swarm optimization (MOPSO) was performed to tie the physiological stresses of the simulated flow field to the measured hemolysis using an equivalent of over one million numerically determined hemolysis predictions. The results show that our optimization is capable of improving upon existing hemolysis models. However, it also unveils some deficiencies and limits of hemolysis prediction with stress-based models, which will need to be addressed in order to improve its reliability.

**Supplementary Information:**

The online version contains supplementary material available at 10.1007/s10237-022-01653-7.

## Introduction

Mechanical circulatory support (MCS) devices are technological innovations that have saved the lives of tens of thousands of diseased people so far (Gregory et al. 2017). The aim of these systems is to maintain blood circulation in the body, in other words, they increase blood pressure in order to compensate for flow resistance and achieve a desired flow rate in the cardiovascular system. Due to the working principles of the MCS devices, supra-physiological stresses of several hundred Pascal can occur (Schüle et al. 2016), which increase the risk of blood trauma. This circumstance requires investigations for hemocompatibility by *in-silico*, *in-vitro*, and *in-vivo* studies in order to improve these systems.

In that regard, flow simulations (*in-silico* studies) are becoming more and more important, especially in the preclinical evaluation of an MCS device (Gregory et al. 2017). Using flow simulations, system designs can be evaluated quickly and cost-effectively regarding their fluid mechanical performance and their blood damage potential. Regarding the latter, the blood flow in the device is computed by the flow simulation and afterward, blood damage is predicted by a blood damage model in the post-processing (Yu et al. 2017).

However, the simulation and blood damage modeling should be carried out in such a trustworthy manner that they reflect reality as close as possible. Only then might flow simulations reduce cost- and resource-intensive *in-vitro* and *in-vivo* studies in the future. This applies, especially to the numerical prediction of blood damage, where studies investigated the degradation of the von-Willebrand factor, platelet activation and subsequent thrombus formation, or hemolysis (Thamsen et al. 2015; Wiegmann et al. 2018; Torner et al. 2019). Many of the applied blood damage models can only be used to make qualitative statements about the damage potential in a MCS device. Moreover, attempts are being made to provide also quantitatively correct results, particularly for hemolysis (Thamsen et al. 2016; Yu et al. 2017; Goubergrits et al. 2019; Nikfar et al. 2020).

The state-of-the-art hemolysis modeling is commonly divided in two model types, the stress-based and strain-based models (Yu et al. 2017). Both couple the viscous stresses in the flow to the occurring hemolysis, but in a different manner. With strain-based models, hemolysis is not a direct result of the stresses alone, but involves and combines intermediate parameters (as the cell deformation, the extension of the cell membrane, transport of hemoglobin through the cell membrane, etc.). The stress-based models relate hemolysis directly to an equivalent stress $\tau _s$ and an exposure time *t* through an empirical power-law relationship (Wu et al. 2019), see Equation (1).

In this study, we focus on the stress-based models for predicting hemolysis. These models are easier to develop and implement in a (commercial) flow solver compared to strain-based models (Goubergrits et al. 2019), and are therefore primarily used in simulative design studies of MCS devices (Taskin et al. 2012; Fraser et al. 2012; Thamsen et al. 2015; Wiegmann et al. 2018). However, the stress-based models are physically more basic than the strain-based models, which raises the question whether a proper hemolysis prediction is possible (on which we will comment later).

1 $$\begin{aligned} H(\tau _s,t) = C \tau _{s}^{\alpha } t^{\beta } \end{aligned}$$

In Eq. (1), the hemolysis value *H* represents the ratio of released plasma-free to the total hemoglobin concentration. Furthermore, a set of empirical constants $(C,\alpha , \beta )$, the so-called power-law constants, are included in that equation. These constants couple the flow variables $\tau _s$ and *t* with the hemolysis value *H*, and are commonly derived from validation experiments in simple blood flows, such as laminar Couette or Couette-Poiselle flows (Giersiepen et al. 1990; Heuser and Opitz 1980; Zhang et al. 2011). In these experiments, hemolysis is determined under defined stresses and exposure times, and correlations are established from these experimental data to create a stress-based hemolysis model. When this methodology is applied to numerical hemolysis prediction, these models are derived in such a way that the numerical hemolysis should agree with measured hemolysis data. However, a large discrepancy between experimentally measured and numerically calculated hemolysis can be observed throughout the literature (a comprehensive review is given in Reference (Yu et al. 2017)). In particular, when hemolysis is computed in MCS devices, it is observed that deviations of more than hundreds of percent could be present between simulative and experimentally assessed hemolysis (Song et al. 2004; Taskin et al. 2012; Myagmar and Day 2015; Wu et al. 2019).

The reasons for the discrepancies are suspected to be - among others - in the experimental model generation, which was mainly done using validation experiments of simple flow configurations in a laminar flow regime in the past (Giersiepen et al. 1990; Heuser and Opitz 1980; Zhang et al. 2011). Here, it is unknown whether these models also have their validity in turbulent flows (Antiga and Steinman 2009) and in the complex flows with compound, three-dimensional flow interactions, as they are present in MCS devices (Torner et al. 2021). Furthermore, the models were defined only for specific stress and exposure time ranges, but they are often applied outside these ranges (Yu et al. 2017).

Because of the points raised, some attempts exist in the literature to improve hemolysis modeling from the numerical side. In these studies, the power-law constants are adjusted to correspond to experimental hemolysis with the help of flow simulations and an optimization algorithm. For example, Fraser et al. (2012) used flow simulations at eight operation points of two MCS devices and a gradient descent algorithm to optimize their numerical hemoylsis results and obtain a new set of power-law constants. Another attempt to find optimized power-law constants for turbulent flows was also proposed by Tobin and Manning (2020). Two test cases (flow simulations in the FDA nozzle and a gauge needle) at six operation points were used to tune the constants $(C,\alpha , \beta )$. Ozturk et al. (2017) developed several modified stress-based model equations with optimized constant sets based on a hemolysis-causing flow variable alternative to shear stress. They fit their model results to experimental hemolysis data by regression analyses and simulations of three flow test cases (Couette, capillary tube, and a jet flow) at several operation points. A different approach was followed by Craven et al. (2019). They optimized the constants $(C,\alpha , \beta )$ specifically for certain devices and blood-donor species by a Kriging surrogate algorithm. In this optimization, analytical solutions of simple shear flows as well as simulations of laminar capillary tube flows were used.

All these literature studies manage to improve hemolysis prediction in their model environment (used flow variables for hemolysis prediction, applied test cases, modified model formulations, etc.). However, none of these newly proposed power-law constants are recognized as a universally valid constant set for stress-based hemolysis prediction so far. For this reason, this study aims to answer the question: *Is it possible to find power law constants* $(C, \alpha , \beta )$ *which predict hemolysis quantitatively correct with the help of a novel optimization algorithm, a commonly used setup for simulating turbulent blood flows and a stress-based hemolysis model?* By commonly used simulation setup, we mean Reynolds-Averaged Navier-Stokes simulations of turbulent blood flows, 1.) where blood is approximated as a single-phase fluid with blood-like flow behavior; 2.) where an equivalent shear stress based on the second invariant of the strain-rate tensor $S_{ij}$ is used for hemolysis prediction; 3.) and where hemolysis is predicted with a stress-based model based on Eq. (1).

Regarding this, the present study aims to find these universally valid constants $(C, \alpha , \beta )$. Therefore, three, well-known test cases from the literature (capillary tube, FDA nozzle, FDA pump) were chosen, all containing a significant portion of turbulent stresses, as they are also present in MCS devices (Torner et al. 2019). A total of 12 operation points were simulated. Afterward, hemolysis was numerically determined and the hemolysis prediction was then optimized using an optimization method based on artificial intelligence, the particle swarm optimization (PSO) (Kennedy and Eberhart 1995). The PSO method has not been used for the optimization of hemolysis prediction so far, but is generally a proper method to find global optimum for equation-based optimization problems (Noel 2012). The hemolysis optimization was performed with a data set containing over one million numerically determined hemolysis results.

## Materials and methods

### Hemolysis modeling and assessment

The computation of hemolysis is one of the most important evaluation aspects for flow analyses in MCS devices (Thamsen et al. 2015; Wiegmann et al. 2018; Wu et al. 2019). Hemolysis computations in complex flow systems can be performed using Lagrangian or Eulerian models, the latter being divided into stress-based and strain-based Eulerian models (Goubergrits et al. 2019). Often, stress-based models are used in nowadays flow simulations (Yu et al. 2017), which originally base on the work by Giersiepen et al. (1990), who related hemolysis to an equivalent stress $\tau _s$ and the exposure time *t* via a power-law, see Eq. (2).

2 $$\begin{aligned} H(\tau _s,t) = \frac{\Delta fHb}{Hb} = C \tau _{s}^{\alpha } t^{\beta } \end{aligned}$$

In this equation, the hemolysis value *H* denotes the ratio between released hemoglobin (increase of plasma-free hemoglobin $\Delta fHb$) and the total hemoglobin (*Hb*) within the blood. The parameters *C*, $\alpha$ and $\beta$ are the empirical constants and Table 1 provides an overview of several commonly used constant sets.

**Table 1:** The table is listing constant sets proposed by various authors (Giersiepen et al. 1990; Heuser and Opitz 1980; Song et al. 2003; Zhang et al. 2011; Fraser et al. 2012). Abbreviations in the first column are Giersiepen and Wurzinger (GW), Heuser and Opitz (HO), Zhang and Taskin (ZT) and Fraser and Zhang (FZ), which are based on the names of the first two authors mentioned in the respective sources

Name	Source	Constants		
		*C*	$\alpha$	$\beta$
GW	Giersiepen et al. (1990)	$3.62\times10^{-7}$	2.416	0.785
HO	Heuser and Opitz (1980); Song et al. (2003)	$1.8\times10^{-8}$	1.991	0.765
ZT	Zhang et al. (2011)	$1.228\times10^{-7}$	1.9918	0.6606
FZ	Fraser et al. (2012)	$1.745\times10^{-8}$	1.963	0.7762

For stress-based Eulerian models, hemolysis can be numerically assessed by linearizing the hemolysis value *H* to $H_L = H^{\frac{1}{\beta }}$ in order to build a transport equation (3), which is solved alongside the flow governing equations.

3 $$\begin{aligned} \frac{{\rm d}H_L}{{\rm d}t} = \frac{\partial H_L}{\partial t} + u_i \frac{\partial H_L}{\partial x_i} = C^{\frac{1}{\beta }} \tau _{s}^\frac{\alpha }{\beta } \end{aligned}$$

If a statistically steady flow is considered, which is a valid assumption for our considered test cases, Eq. (3) can be simplified. Then, hemolysis can be assessed globally in a flow domain (inflow-to-outflow) in order to avoid the necessity of computing an extra transport equation. The exact derivation of this simplified hemolysis model will not be explained here, but is further outlined in Garon and Farinas (2004). A linearized hemolysis value $H_L$ is computed (Eq. (4)) as an outcome of the model simplification, which base on a integral examination of the equivalent stresses $\tau _s$ in the flow domain and the flow rate *Q*. It will be shown in Sect. 3.1 that both, Eqs. (3) and (4), will lead to similar numerical hemolysis results for the investigated flow cases in this paper.

4 $$\begin{aligned} H_L = \frac{1}{Q} \iiint \limits _{V} C^{\frac{1}{\beta }} \tau _{s}^\frac{\alpha }{\beta } \, {\rm d}V \end{aligned}$$

Numerically, the results of Eqs. (3) and (4) can be converted by Eq. (5) into a key parameter for hemolysis assessment - the *modified index of hemolysis* *MIH*.

5 $$\begin{aligned} {\rm MIH_{num}} = H_{L}^\beta\cdot10^6 \end{aligned}$$

This index is also used to evaluate the occurring hemolysis by experiments. The experimental ${\rm MIH_{exp}}$ (Eq. (6)) is defined, e.g., by the ASTM F1841-97 standard (ASTM 2017). Determining ${\rm MIH_{exp}}$ requires the measurement of the plasma-free hemoglobin $\Delta fHb$, the blood hemoglobin concentration *Hb*, the hematocrit $H_{\rm ct}$, as well as the flow rate *Q*, the total time $\Delta t$ and the total volume of blood *V* pumped through a closed circuit.

6 $$\begin{aligned} {\rm MIH_{exp}} = \frac{1}{Hb}\frac{\Delta fHb}{\Delta t} \frac{V}{Q} (1 - H_{ct}) 10^6 \end{aligned}$$

### Equivalent stresses $\tau _s$

The surface stresses working upon a fluid element within a flow can be defined by the sum of isometric and deviatoric contributions. The latter describes the friction stresses within the fluid and is characterized by the viscous stress tensor $\tau _{ij}$ in Eq. (7).

7 $$\begin{aligned} \tau _{ij} = 2\mu S_{ij} \end{aligned}$$

In Eq. (7), $\mu$ represents the viscosity and $S_{ij}$ the strain rate tensor, which is defined in Eq. (8) and describes the rate of deformation of a fluid element in an incompressible flow.

8 $$\begin{aligned} S_{ij} = \frac{1}{2}\left(\frac{\partial u_i}{\partial x_j} + \frac{\partial u_j}{\partial x_i}\right) \end{aligned}$$

The viscous stress tensor $\tau _{ij}$ forms the basis of most stress-based hemolysis models. However, for complex flow fields often a scalar notation is preferred, which can be derived from the second invariant of the strain rate tensor (Bludszuweit 1995; Hund et al. 2010), as shown in Eq. (9).

9 $$\begin{aligned} \tau _{s} =\mu \sqrt{2 S_{ij} S_{ij}} \end{aligned}$$

In order to account for the whole equivalent stresses of a simulated, turbulent blood flow by solving the commonly used Reynolds-Averaged Navier-Stokes (RANS) equations, initially Hund et al. (2010), and later Wu et al. (2019) and Konnigk et al. (2021) defined a equivalent stress $\tau _{\rm s,eff}$, which also accounts for the non-resolved, modeled turbulent contribution of the equivalent stresses, see Eq. (10). In that equation, the overbar in $\overline{S_{ij}S_{ij}}$ denotes the time-average of the tensor multiplication $S_{ij}S_{ij}$, while $\varepsilon _{\rm mod}$ represents the modeled turbulent dissipation rate from the turbulence model and $\rho$ is the density of the fluid.

10 $$\begin{aligned} \overline{\tau }_{\rm s,eff} = \sqrt{ 2 \mu ^2 \overline{S_{ij}S_{ij}} + \rho \mu \varepsilon _{\rm mod} } \end{aligned}$$

### Particle swarm optimization (PSO)

The *particle swarm optimization* was discovered through the simulation of a simplified social model of animal swarms (i.e., bird flocks and fish schools) and was first introduced by Kennedy and Eberhart (1995).

In all PSO algorithms, the movement of the particles is a function of three elements: the current velocity *v* and weight of the particle (i.e., particle inertia), the location of the personal best value *p* achieved by the particles in previous iterations and the location of the global best value *g* of the entire swarm. This basic principle is governed by the following two equations, Eqs. (11) and (12).

11 $$\begin{aligned} v_{i,t+1}\,\,=\;\,\, & {} \omega v_{i,t} + c_1r_1 (p_{i,t} - x_{i,t}) + c_2 r_2 (g_{i,t} - x_{i,t}), \end{aligned}$$

12 $$\begin{aligned} x_{i,t+1}\,\,=\,\, & {} x_{i,t} + v_{i,t+1}, \end{aligned}$$

where *t* being the current iteration step index, $\omega$ representing the inertia weight, $c_1$ and $c_2$ being the cognitive and social learning factors, respectively, and $r_1$ and $r_2$ are random numbers between zero and one in the equations above. Clerc and Kennedy (2002) introduced a constriction coefficient which ensures convergence by linking the $\omega$ and *c* using the intermediate parameter $\varphi > 2$ as shown in Eqs. (13) and (14). The learning factors are merged into one, $c=c_1=c_2$, in order to simplify the update equations.

13 $$\begin{aligned} \omega\,\,=\,\, & {} \frac{1}{\varphi -1+\sqrt{\varphi ^2-2\varphi }} \end{aligned}$$

14 $$\begin{aligned} c\,\,=\,\, & {} \omega \varphi \end{aligned}$$

In the current study, an extended version of the PSO is used, the *multiple objective particle swarm optimization* (MOPSO). It was firstly proposed by Coello et al. 2002, and uses the PSO to solve a pareto optimization problem. The algorithm tries to find the optimal compromise between multiple criteria. The optimization results are presented as the repository particles, which are the dominant particles, i.e., solutions that can not be improved without worsening the result for any of the other criteria.

**Fig. 1:**
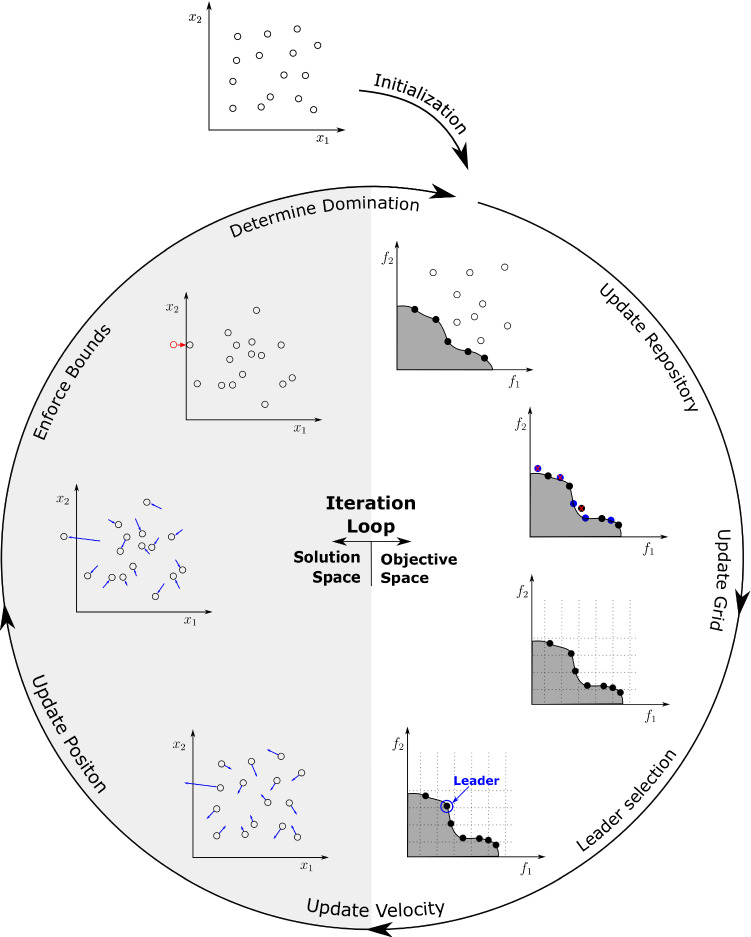
Overview of the MOPSO algorithm. Particles are randomly placed into the solution space and moved in an iteration loop. The iteration loop can be split into two sides: the objective and the solution space. The former uses the fitness values to determine the Pareto front (stored in the repository), upgrade the hypercube-grid and select the leader (global best) and local best position. The global and local leader positions *g* and *p* are then used to determine the motion in the solution space using Eqs. (11) and (12)

The MOPSO algorithm used in this study was coded and computed in *Matlab 2020b* (The Math Works, Inc., MA , USA). It is based on the implementation written by Heris (2015), but significantly altered to be used for this specific task. The algorithm is visualized in Fig. 1 and can be split into two phases, the initialization phase and the iteration loop. The first phase initializes the particles randomly into the domain and provides them with randomized velocity vectors. This phase also determines the first fitness values, creates the hypercube grid and stores the first particles into the repository. The iteration loop then updates these parameters, using Eqs. (11) and (12), until the maximum number of iterations has been reached. Particles that move outside of the domain boundaries are forced back on the domain edges but their velocity is maintained. When the repository is full, random particles are removed from the hypercubes with the highest population, in order to ensure a relative evenly defined Pareto-front. Finally, it should be mentioned that no additional mutation is used for the position updates, as is the case with Heris’ algorithm.

The *MIH* values are evaluated for the MOPSO by exporting finite volumes $\Delta V$ and the equivalent stresses $\overline{{\tau }}_{s,eff}$ from the simulation results of the flow solver *ANSYS CFX* to *Matlab*. This allows the entire optimization to be performed in *Matlab* using Eq. (17) as an approximation of Eqs. (4) and (5). The new equation can be derived using typical finite volume approximations of an arbitrary variable $\phi$, Eqs. (15) and (16), to solve the volume integral. By decoupling the optimization process from the flow simulation, the overall simulation and evaluation time is reduced significantly without reducing the accuracy of the hemolysis prediction. It will be shown in Sect. 3.1 that the hemolysis prediction in *Matlab* will give similar results as the equivalent one performed in *ANSYS CFX*.

15 $$\begin{aligned} \iiint \limits _{V} \phi \, {\rm d}V= & {} \sum _{k}\iiint \limits _{V_k} \phi \, {\rm d}V \end{aligned}$$

16 $$\begin{aligned} \sum _{k} \iiint \limits _{V_k} \phi \, {\rm d}V= & {} \sum _{k} \overline{\phi } \Delta V_k \approx \sum _{k} \phi _p \Delta V_k \end{aligned}$$

17 $$\begin{aligned} {\rm MIH_{num}}= & {} \big [\frac{1}{Q} C^{\frac{1}{\beta }} \sum _{k} ( \tau _{s,k}^\frac{\alpha }{\beta } \, \Delta V_k ) \big ]^\beta\cdot10^6 \end{aligned}$$

The specific algorithm parameters used for the optimization are listed in Table 2. Two optimization domains were used in this study. One is the literature domain, a range in which the literature from Table 1 defines the constants $(C,\alpha ,\beta )$ so far. The other is a mathematical domain that allows a wide range of $(C,\alpha ,\beta )$. Here, the limits of the constants were arbitrarily extended by multiple orders of magnitude to potentially find the optimal constant set in this wider range.

**Table 2:** Algorithm parameters of the two individual optimization runs. $N_p$ represents the number of particles, $N_r$ represents the repository capacity, $t_{\rm max}$ is the maximum amount of optimization iterations, $\varphi$ is used to determine the inertia weight and learning factors, *UB* and *LB* are the upper and lower boundaries, $N_g$ is the number of hypercubes in each dimension

Domain	Parameters	
	Mathematical range	Literature range
$N_p$	100	100
$N_r$	1000	1000
$t_{\rm max}$	1000	1000
$\varphi$	2.01	2.01
$UB-[C,\alpha ,\beta ]$	$[ 10^{-5}, 20.0, 20.0]$	$[ 10^{-7}, 2.5, 1.0]$
$LB-[C,\alpha ,\beta ]$	$[10^{-30}, 0.1, 0.1]$	$[ 10^{-9}, 1.5, 0.5]$
$N_g$	50	50

Two statistical functions will be used as fitness criteria by the MOPSO algorithm. The first will be an representation of the relative mean error, Eq. (18), among all considered operation points ($N=12$) compared to the experimental hemolysis values at a specific operation point. However, in order to account for the measurement uncertainties in the experiments (${\rm MIH_{exp,min}}$ and ${\rm MIH_{exp,max}}$), the error will be set to zero when ${\rm MIH_{num}}$ lies within these uncertainties, as shown in Eq. (19). In return, the error is larger than zero, when ${\rm MIH_{num}}$ deviates from the experimental hemolysis result, and could be up to one, when ${\rm MIH_{num}/MIH_{exp}}$ reaches either zero or infinity. A written and visual explanation of the error estimation based on equation (19) can be found in the Supplementary file 1.

18 $$\begin{aligned} \eta (\xi _i)= & {} \frac{1}{N} \sum _{i=1}^{N} \xi _i \end{aligned}$$

19 $$\begin{aligned} \xi _i= & {} {\left\{ \begin{array}{ll} \left| \frac{1}{\left| ln\left( \frac{{\rm MIH_{num}}}{{\rm MIH_{exp}}}\right) \right| +1 }-1 \right| &{} \text {for} \ {\rm MIH_{num}< MIH_{exp,min}} \ \text {or} \ {\rm MIH_{num} > MIH_{exp,max}}\\ 0 &{} \text {for} \ {\rm MIH_{exp,min}< MIH_{num} < MIH_{exp,max}} \\ \end{array}\right. } \end{aligned}$$

Finally, a linear correlation coefficient is used to define a value between 0 and 2, where the former equals a perfect correlation and the latter a negative correlation. It is based on the Pearson correlation coefficient where $A_i$ and $B_i$ represent the sample points.

20 $$\begin{aligned} r(A_i,B_i) = \Bigg | \frac{1}{N-1} \sum _{i=1}^{N} \big ( \frac{A_i - \eta _A}{\sigma _A} \big ) \big ( \frac{B_i - \eta _B}{\sigma _B} \big ) - 1\Bigg | \end{aligned}$$

Together, the two functions $\eta (\xi _i)$ and $r(A_i,B_i)$ define the optimization objectives of the MOPSO algorithm, as shown in Eq. (21).

21 $$\begin{aligned} \text {min} \ f_i = \{\eta , r\} \end{aligned}$$

In order to evaluate the repository to find the best optimized constant set $(C, \alpha . \beta )$, the particles can be ranked by calculating the overall fitness value $F_n$, see Eq. (22). A fitness value of $F_n=0$ reflects a perfect match between the numerically and experimentally assessed hemolysis values.

22 $$\begin{aligned} F_n = \frac{\eta + r}{2} \end{aligned}$$

### Test cases, operation points and simulation setup

Various numerical simulations were carried out for the test cases at specific operation points (OPs). All simulated OPs were employed for the MOPSO. Before the details of the respective test case are presented, the commonalities will be mentioned here. All simulations were computed using Reynolds-averaged Navier-Stokes equations (RANS, unsteady RANS for the FDA pump) by the commercial flow solver *ANSYS CFX* (Ansys, Inc., PA, USA). A second-order upwind scheme was used for the spatial discretization. Turbulence was modeled using the *k*-$\omega$-SST-model (Menter 1994). Additionally, a $\Gamma$-$\Theta$ transition (Menter et al. 2006) and curvature correction model (Smirnov and Menter 2009) were applied. The hexahedral, block-structured meshes were created with *ANSYS ICEM CFD*. All test cases were simulated in the way as turbulent blood flows, e.g., in MCS devices, are commonly computed. Therefore, blood was treated as a Newtonian, single-phase fluid, which properties were specified as stated in the experiments.

#### Test case 1: FDA nozzle

**Fig. 2:**
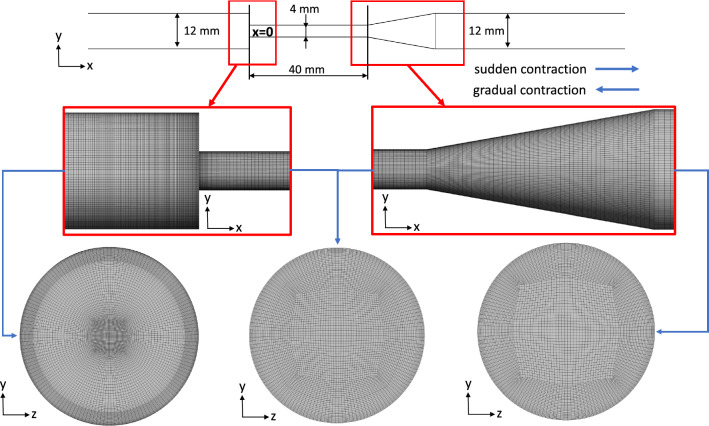
Illustration of the FDA nozzle case. The grid in the nozzle, diffusor and throat section is shown in the subfigures

The first test case is the benchmark FDA nozzle model presented by Hariharan et al. 2011 or Herbertson et al. 2015. Figure 2 and Table 3 illustrate and summarize the geometry and flow conditions. Flow measurements of the FDA nozzle were made and are accessible via a data repository (Hariharan and Malinauskas 2017). Two nozzle geometries with a sudden and a gradual contraction were used. We will refer to these OPs as VGC and VSC (validation of gradual resp. sudden contraction) in the following.

Flow-induced hemolysis was evaluated in three OPs (Herbertson et al. 2015). In one of the three OPs, the flow goes through the gradual cone entrance, while the other two flow through the device in the opposite direction (sudden contraction entrance). Henceforth, the first OP with a flow rate of 6 l/min will be referred to as 6LGC, while the latter ones with flow rates of 5 l/min and 6 l/min will be named 5LSC and 6LSC, respectively.

**Table 3:** Flow conditions for the 5 OPs of the FDA nozzle test case. Two will be used as simulation validation, while the other three are used for the optimization algorithm. The Reynolds number $Re_{\rm nozzle}$ refers to the condition at the narrowest point

	Validation		Optimization		
	VGC	VSC	5LSC	6LGC	6LSC
				
*Q*[ l/min]	4.06	4.06	5.05	5.94	6.07
$\mu$ [mPas]	3.5	3.5	4.21	4.21	4.21
$\rho$ ${\rm [kg/m^3]}$	1056	1056	1040	1040	1040
$Re_{\rm nozzle}$	6500	6500	6650	7860	8020
${\rm MIH_{exp}}$	-	-	$0.29\pm 0.25$	$0.02\pm 0.13$	$1.24\pm 0.67$
*fluid*	${\rm blood-analogous}$ fluid		bovine blood with $H_{ct}\approx 36\%$		

#### Simulation setup for the FDA nozzle

The final grids consist of approximately 5.0*M* elements for the VSC, 5LSC and 6LSC, and 8.2*M* elements for the VGC and 6LGC operation points. Different grid resolutions for the respective OPs (SC or GC) were necessary since the verification (Sect. 3.1) indicated verified results at different grid sizes for SC and GC. Verification was done for VGC and VSC using an advanced grid convergence study introduced by Eca and Hoekstra (2014), for which up to six coarser grids were created with a grid coarsening factor of 1.25. A maximal wall distance of $y^+_1\approx 3$ with a near-wall growth rate of 1.1 was ensured. Furthermore, aspect ratios were smaller than 60, volume changes less than five and grid angles greater than $34^{\circ }$. Since the inflow Reynolds number is small and no disturbances are expected at the domain inlet, a laminar Hagen-Poiseulle velocity profile was imposed at the inlet.

#### Test case 2: FDA blood pump

**Fig. 3:**
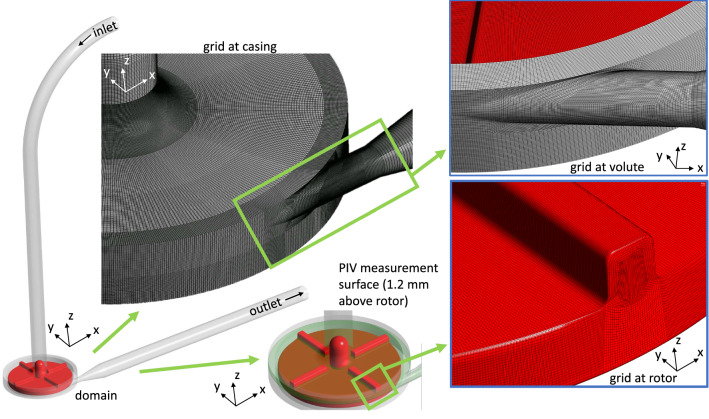
Illustration of the FDA pump test case. The grid at the casing, volute and rotor is shown in the subfigures. Furthermore, the position of the velocity measurement plane for validation is sketched in one subfigure

The second test case is the FDA blood pump (Malinauskas et al. 2017), which is shown in Fig. 3. In total, six operation points on two characteristic curves were carried out, covering flow rates between $Q=(2-7)~{\rm l/min}$ at two rotational speeds $n=2500$ rpm and $n=3500$ rpm. In this study, five of the six operation points (OPs) were simulated. The flow conditions of the five OPs are summarized in Table 4. Velocity measurements were carried out with a blood-analogous fluid, whilst pressure head $H_p$ and plasma-free hemoglobin $\Delta fHb$ measurements are conducted with porcine blood. For determination of ${\rm MIH_{exp}}$, the total blood hemoglobin concentration *Hb* must be specified, but was not provided by Malinauskas et al. (2017). Hence, it had to be estimated using the measured hematocrit and the relationship of Briggs and Bain (Lewis et al. 2017). From this, we determined $Hb=12,000~{\rm mg/dl}$.

**Table 4:** Flow conditions for the 5 OPs of the FDA blood pump. Hemolysis and pressure heads were determined for all cases and velocities were measured for OP1, OP4 and OP5. Results of OP1 were used for a grid convergence study

	OP1	OP3	OP4	OP5	OP6
*Q* [l/min]	2.5	4.5	6.0	6.0	7.0
*n* [rpm]	2500	3500	2500	3500	3500
$\mu _{\rm blood}$ [mPas]	3.4	3.4	3.4	3.4	3.4
$\rho _{\rm blood}$ ${\rm [kg/m^3]}$	1030	1030	1030	1030	1030
${\rm Re_{p}=\rho \omega D^2 / \mu}$	209, 338	293, 073	209, 338	293, 073	293, 073
*Calculated* $MIH_{exp}$	$0.80 \pm 1.28$	$1.24 \pm 0.97$	$0.78 \pm 0.59$	$0.77 \pm 0.47$	$2.44 \pm 1.89$
*Fluid*	${\rm blood-analogous}$ fluid (PIV) & porcine blood ($H_p$, hemolysis)				

#### Simulation setup for the FDA blood pump

The final grid for the simulation had a size of 24.3 million elements, with a maximal aspect ratio around 1000 and a minimal grid angle of $23^\circ$. The near-wall grid was built to reach $y_1^+\approx 1$ in all OPs with wall-normal growth rate of 1.3. Parts of the computational grid are displayed in Fig. 3. The grid was verified by a grid convergence study developed by Roache (1998) at OP1 using two coarser grids (coarsening factor of 1.25).

The rotor rotated at speeds defined in Table 4. Transient rotor-stator coupling was used with a time step equal to $5^{\circ }$ rotation of the rotor. A total of 20 revolutions were calculated. Convergence was reached, when RMS residuals dropped at $10^{-4}$ and all monitored values were in a statistically steady condition. Time averaging was done for ten revolutions. A constant flow rate was given at the outlet, while a zero, total pressure was set at the inlet. Since the inflow pipe Reynolds number does not clearly indicate a laminar or turbulent state ($Re_{pipe} \le 3700$), a low turbulence intensity of $1\%$ and eddy viscosity ratio $\nu _t/\nu =1$ were specified at the inlet.

#### Test case 3: capillary tube

**Fig. 4:**
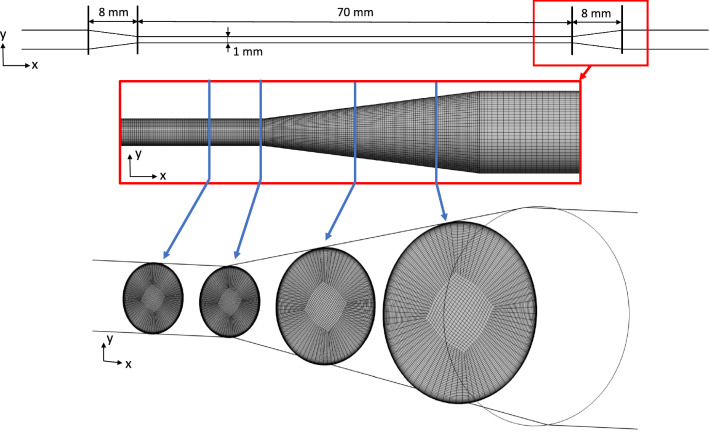
Illustration of the capillary tube test case with parts of the grid in the two subfigures

The third test cases were derived from hemolysis experiments in a capillary tube (index: ct) used by Kameneva et al. (2004) and is sketched in Fig. 4. Flow-induced hemolysis in both laminar and turbulent conditions were measured. The turbulent flow conditions were considered in this study and are listed in Table 5. A suspension of washed bovine red blood cells in saline were used. Beside hemolysis measurements, the authors also determined the friction value $\lambda _{\rm turb}$, which was determined by pressure measurements and Eq. (23). This $\lambda$-value was also used in the present study as validation. The total blood hemoglobin concentration *Hb* was not provided for this test case, but is needed for ${\rm MIH_{exp}}$, and can be be estimated by the measured hematocrit as it was also done in Wu et al. (2019) and Craven et al. (2019). Hence, we determined $Hb=8000~{\rm mg/dl}$.

23 $$\begin{aligned} \lambda _{\rm turb} =0.316/Re^{0.25} \end{aligned}$$

**Table 5:** Flow conditions of the capillary tube case. Hemolysis and friction values $\lambda$ were determined, and a verification was made for all OPs. The Reynolds number $Re_{\rm ct}$ refers to the conditions at the narrowest point

	ct2230	ct3500	ct4500	ct5100
*Q* [l/min]	0.21	0.33	0.42	0.48
$\lambda$ $[-]$	0.0446	0.0417	0.0390	0.040
$\mu$ [mPas]	2.0	2.0	2.0	2.0
$Re_{\rm ct}$	2230	3500	4500	5100
Calculated ${\rm MIH_{exp}}$	$8.7 \pm 5.9$	$9.4 \pm 4.9$	$37.9 \pm 10.0$	$85.5 \pm 19.5$
Fluid	Bovine blood with $H_{ct}=24\%$ ($\lambda _{turb}$, hemolysis)			

#### Simulation setup for the capillary tube

The final grid had a size of 6.5 *M* grid elements with minimal grid angles of $50^{\circ }$, aspect ratios of $\le 100$ and a maximal volume change of four. The wall-layers were meshed with a growth rate of 1.2 and $y^+_1\le 0.6$. The final grid is displayed in Fig. 4. A convergence study developed by Roache (1998) was performed using two additional grids (coarsening factor of 1.45). Laminar Hagen-Poiseuille velocity profiles were specified at the inlet boundary, whilst a zero pressure was assumed for the outlet.

## Results

### Verification and validation

*FDA nozzle*

**Fig. 5:**
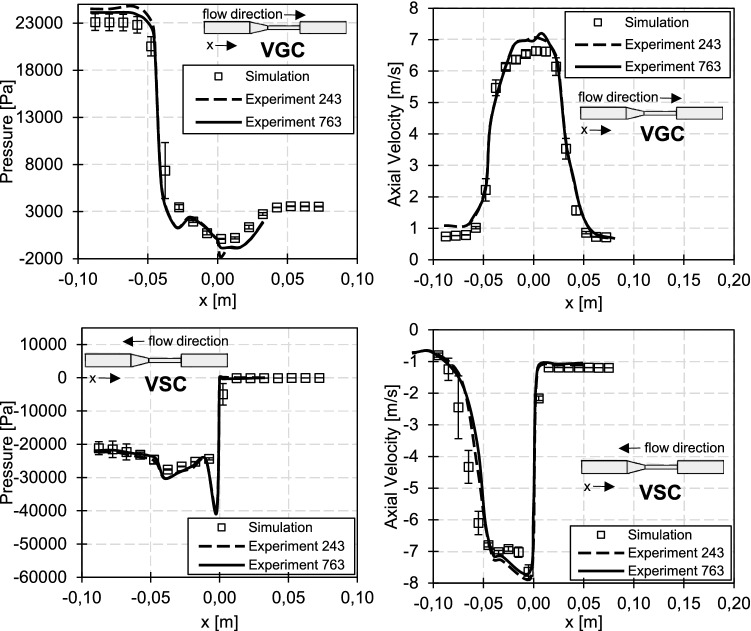
Verification and validation of the FDA nozzle case. The error bars reflect the uncertainty due to the spatial discretization error. The results with the finest grid are compared with experiments from Hariharan and Malinauskas (2017)

Figure 5 shows the verification and validation of the OPs - *VGC* and *VSC*. The pressures and velocities follow the measurements with small uncertainties in the inflow region. Just in regions with large gradients, higher uncertainties are observable. Nonetheless, all simulation results indicate that the flow physics within the nozzle can be accurately reproduced.

*FDA pump*

**Fig. 6:**
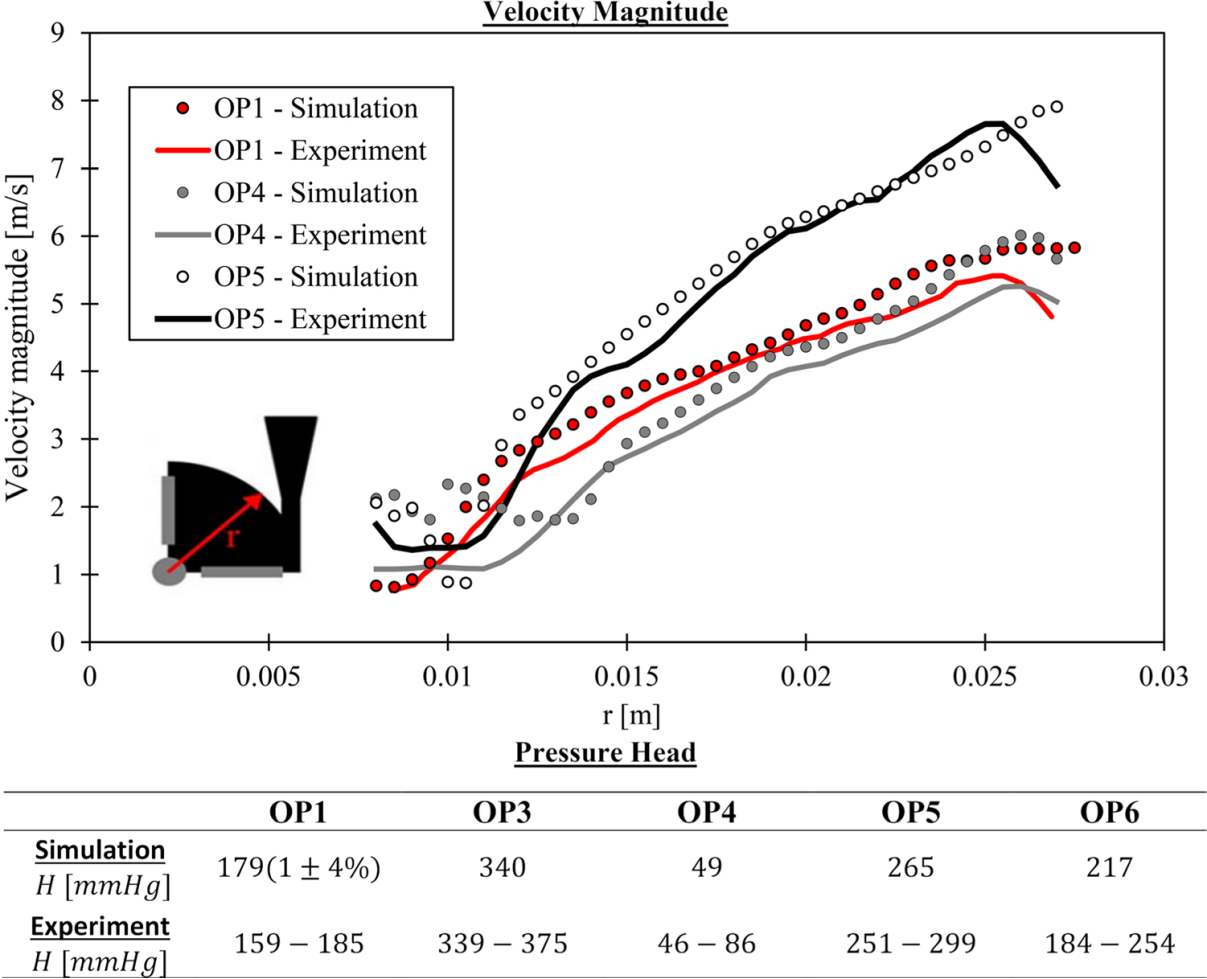
Verification and validation of the FDA pump case. Experiments are taken from Hariharan et al. (2018). Top: Velocities in the rotor and volute. Bottom: Pressure heads for all OPs. The percentage uncertainty due to the spatial discretization is displayed for OP1

The comparison between simulations and experiments assessed results are displayed in Fig. 6. A close agreement in the velocities can be observed for all OPs. Also, the pressure heads are within the measured ranges. Furthermore, the influence of grid errors is reasonably small with $\pm 4~\%$ uncertainty for OP1.

*Capillary tube*

The friction coefficients $\lambda _{turb}$ are shown in Table 6. It can be seen that the uncertainties (percentage values in the brackets) is negligible with a maximal uncertainty of $\pm 0.56\%$. Also, the friction losses are similar in simulation and experiment with deviations between $-10.0\%$ and $+4.8\%$.

**Table 6:** Verification and validation of the capillary tube case. The percentage uncertainty in the simulation data represents the spatial discretization error. The experimental data are taken from Kameneva et al. (2004)

OP	Friction coefficient $\lambda _{turb}$			
	ct2230	ct3500	ct4500	ct5100
*Simulation*	0.042	0.044	0.038	0.036
$\lambda _{turb}~[-]$	$(1\pm 0.10\%)$	$(1\pm 0.56\%)$	$(1\pm 0.23\%)$	$(1\pm 0.13\%)$
*Experiment*	0.045	0.042	0.039	0.040
$$\lambda _{turb}~[-]$$				
*Rel. Deviation*	$-6.7\%$	$+4.8\%$	$-2.6\%$	$-10.0\%$

*Verification of the process chain*

In order to verify the process chain from simulation results to optimization results, numerical hemolysis was obtained for all OPs of the test cases by three different ways. The aim is to show that the *MIH*, determined by the Eulerian transport equation (Eqs. (3), (5) and (10)), reflects similar hemolysis for a statistically steady flow as an *MIH*, determined by the volume integral (Eqs. (4), (5) and (10)). Both results are obtained in the flow solver *ANSYS CFX*. The *MIH* based on the volume integral was then used for the optimization in *Matlab*. Therefore, Eq. (17) has been derived, which should reflect identical hemolysis results in *Matlab* as the volume integral in *ANSYS CFX*. In Table 7, the computed *MIH* are displayed for one representative OP for each test case. As can be seen, the computation by the different ways results in similar values. Only for the FDA pump, there are slight discrepancies of $\approx 1\%$ between the results of the transport equation and the volume integral. Nevertheless, the small deviations seem justifiable in the context that the uncertainties in ${\rm MIH_{exp}}$ are much higher than these numerical deviations.

The advantage of MIH calculation in *Matlab* can be seen when comparing the computing times between the different approaches. While the pump simulation must be run for about 130 revolutions (several days computing time with 160 processors) for the evaluation of the transport equation, the volume integral in Matlab can be calculated in a tenth of a second. Therefore, the MIH calculation in *Matlab* allows a fast hemolysis prediction and a data set of over one million of numerically determined hemolysis results for the MOPSO algorithm.

**Table 7:** Verification of the process chain from the flow solver *ANSYS CFX* to *Matlab* using different ways of *MIH* calculation. The results of one representative OP are shown for each test case. The constant set $(C, \alpha , \beta )$ of (HO) Heuser and Opitz (1980) was used. Please note that all calculations were performed with the stresses defined in Eq. (10)

Variable	Equation	Program	FDA nozzle	FDA pump	Cappilary tube
			6LGC	OP1	ct4500
${\rm MIH_{num}}$	(3) & (5)	*ANSYS CFX*	7.0	8.3	12.2
${\rm MIH_{num}}$	(4) & (5)	*ANSYS CFX*	7.0	8.2	12.2
${\rm MIH_{num}}$	(17)	*Matlab*	7.0	8.2	12.2

**Fig. 7:**
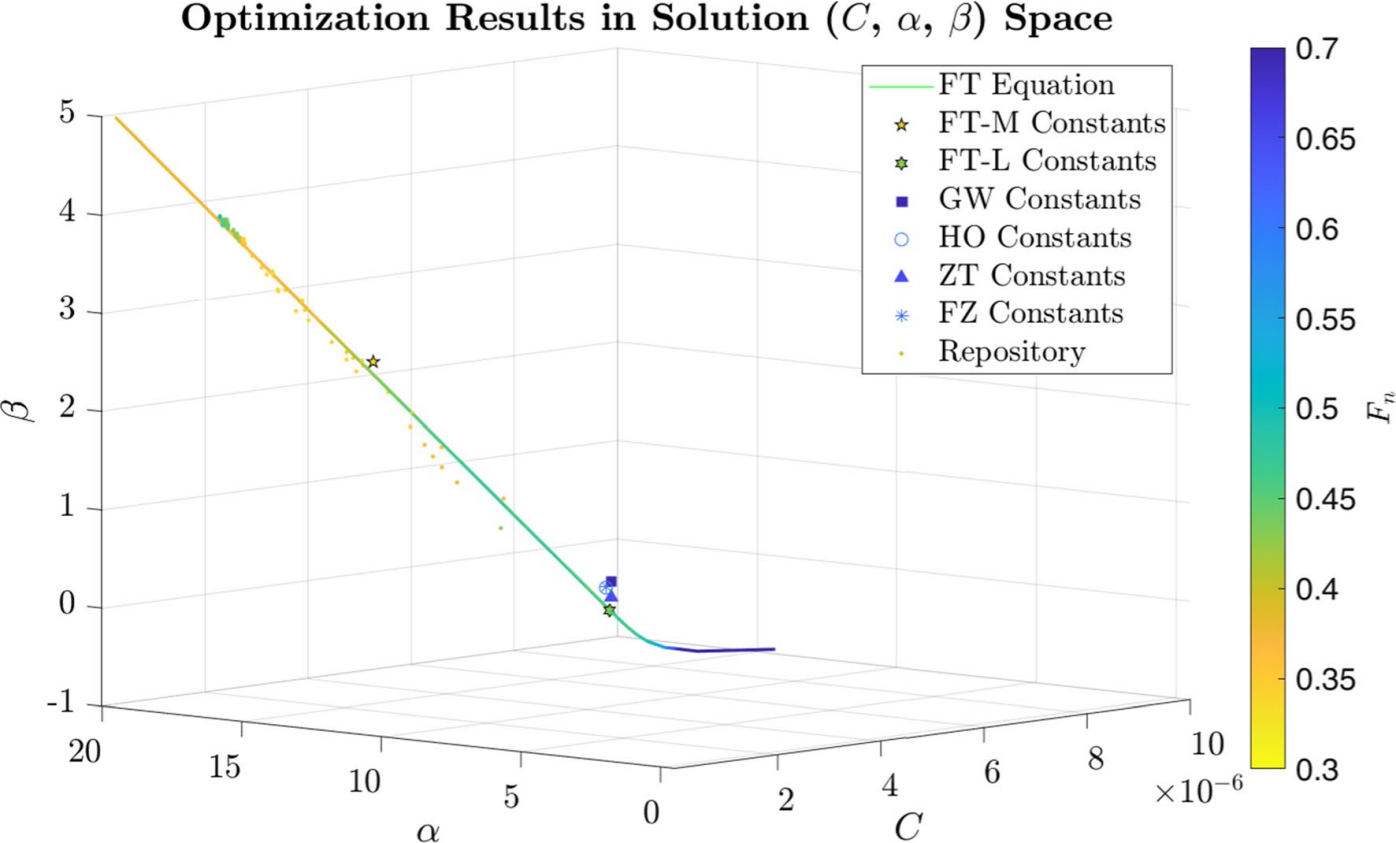
Solution space of the MOPSO results. The line indicates a linear equation, which approximates the best optimization results. Also, the constant sets from the literature (Table 1) are included

### Multi-objective particle swarm optimization (MOPSO)

The optimized constant sets $(C, \alpha , \beta )$ from the combined repository of both MOPSO runs (mathematical and literature range) are displayed in Fig. 7. For every optimized constant set (also called particle), the fitness values $F_n$ based on Eq. (22) are also indicated. Furthermore, Fig. 7 shows the positions and fitness values of the constants from the literature (Table 1). It is noticeable from the MOPSO results that the best particles with the smallest fitness values ($F_n<0.5$) are not at a certain spot, but are distributed in the solution space in a defined pattern. This pattern can be approximated by a three-dimensional linear equation (FT equation), with the help of principal component analysis:

24 $$\begin{aligned} \begin{pmatrix} ln(C) \\ \alpha \\ \beta \\ \end{pmatrix} = \begin{pmatrix} -25.6767 \\ 3.2650 \\ 0.9065 \\ \end{pmatrix} + s \begin{pmatrix} -0.9638 \\ 0.2584 \\ 0.0652 \\ \end{pmatrix}. \end{aligned}$$

**Table 8:** Fitness values and constants of the MOPSO results and the literature. FT (Frank-Torner) is the best constant set from the MOPSO in the mathematical (FT-M) and literature (FT-L) range. GW stands for Giersiepen-Wurziger (1990), HO for Heuser-Opitz (1980), ZT for Zhang-Taskin (2011) and Fraser-Zhang (2012) . Note, the table contains both the values for the unmodified Pearson correlation coefficients *R* and the modified variant *r* (Eq. (20)). Additionally, the *NiB* (Number in Bounds) variable denotes the amount of OPs, where values lie within the experimental uncertainties

	Hemolysis constants					
	$FT-M$	$FT-L$	*GW*	*HO*	*ZT*	*FZ*
*C*	$4.256\times10^{-24}$	$1.000\times10^{-9}$	$3.62\times10^{-7}$	$1.800\times10^{-8}$	$1.228\times10^{-7}$	$1.745\times10^{-8}$
$\alpha$	10.2860	1.8277	2.416	1.991	1.9918	1.963
$\beta$	2.8073	0.5392	0.7850	0.765	0.6606	0.7762
$\eta$	0.34	0.32	0.86	0.58	0.75	0.57
*r*	0.32	0.53	0.53	0.62	0.55	0.64
NiB	7	7	0	2	0	2
$F_n (\eta , r)$	0.33	0.43	0.69	0.60	0.65	0.61
$R_{\rm all}$	0.68	0.47	0.47	0.38	0.45	0.36
$R_{\rm ct}$	0.99	0.90	0.92	0.90	0.90	0.90
$R_{\rm nozzle}$	0.99	0.99	0.99	0.91	0.98	0.88
$R_{\rm pump}$	0.40	0.47	0.44	0.43	0.44	0.43

In the range of $s = [-10; 70]$, the constant sets resulting from this equation all have a fitness value $F_n < 0.5$. The constants from the literature are close, but not directly on the line of the FT equation in Fig. 7. This is also indicated by the greater fitness values $F_n>0.5$ of these constant sets compared to the best MOPSO results, which are shown in Table 8. Here, the fitness values of the two best particles from the mathematical (FT-M) and literature (FT-L) range of the MOPSO are displayed together with the fitness values from the literature constants (GW, HO, ZT, FZ). The best constants from the MOPSO are called FT (Frank-Torner) constants for consistency with the other names of the constant set in the text. The fitness values of the FT constants are significantly reduced to $F_n=0.33$ (FT-M) and $F_n=0.43$ (FT-L) compared to the lowest fitness value $F_n=0.6$ of the literature constants (HO). In addition, it can be seen that even the MOPSO results do not reach the zero fitness value that would be present if all numerical hemolysis predictions were consistent with the experiments.

**Fig. 8:**
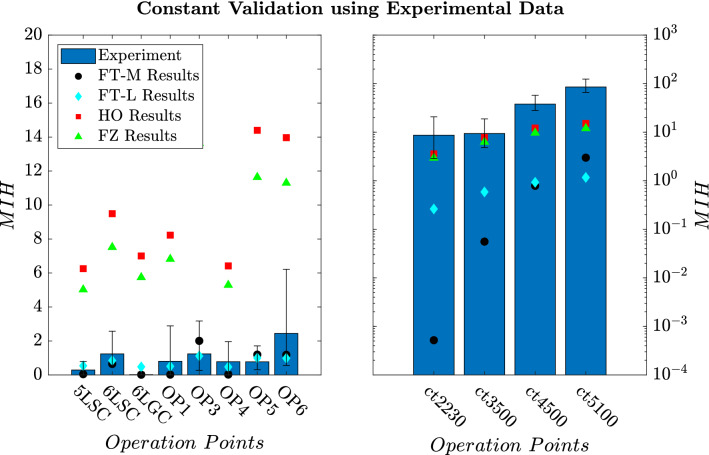
*MIH* results of the test cases. The experimentally assessed hemolysis is shown as well as the numerical predicted hemolysis from the best constant sets based on Table 8

These deviations of numerics and experiments are noticeable in Fig. 8, where the hemolysis predictions are shown for all operation points and test cases. The experimental results are displayed as bars with their uncertainty interval, and the numerical results with the best constants (FT-M, FT-L, HO, FZ) are indicated by dots. For the FDA test cases (nozzle and pump), hemolysis predicted with the FT-M and FT-L constants is closer to the experimental hemolysis results compared to the hemoylsis results obtained with the literature constants. Nonetheless, all constants fail to predict the hemolysis of the capillary tube flow (ct) at high Reynolds numbers. Hemolysis predicted with the HO constants agrees best with the experimental hemolysis for the capillary tube, but is still one order of magnitude lower than the experimental results at *ct5100*.

It should be noted that the hemolysis from the MOPSO models is able to lie within the uncertainty interval of the experimental hemolysis for 7 of the 12 OPs ($NiB=7$), while the literature models only hit a maximum of two (HO and FZ).

## Discussion

The present paper aimed to answer the question: *Is it possible to find universal power law constants* $(C, \alpha , \beta )$ *using an optimization algorithms in combination with flow simulations and stress-based models for hemolysis prediction?*

In an attempt to answer this question, dozens of MOPSO runs were performed, together evaluating millions of numerically predicted hemolysis results. The findings of this study fail to provide a conclusive answer to this question. However, it was able to find improvements and has unveiled some interesting observations, which invite a discussion in a broader context.

In the presented opmitization as well as in preliminary MOPSO tests, it became apparent that it was possible to optimize the OPs individually, but not collectively. The individual optimization in preliminary tests showed that there are multiple constant sets that are able to perfectly match the experimental MIH at a specific operation point. This indicates that the algorithm is generally able to find universal constants, but invites the question, why a collective optimization is seemingly impossible.

Also the optimization with all 12 OPs indicates an multi-modal nature (multiple global optima). This nature resulted in a mathematical relation for the best constant sets, which is expressed by the 3D linear Frank-Torner (FT) equation (24). The constants from the current literature are relatively close to this equation, but perform worse than the best constants derived from the presented MOPSO, which are closer to the line.

The two best performing constant sets of the whole optimization problem were FT-M and FT-L. The former is the best model found in the whole optimization. The latter is more suited for flow simulations in flow solvers due to the floating point limitations, when hemolysis is predicted in the flow solver by Eq. (3) or Eq. (4). The constant set of FT-M has an overall better agreement with the experimental data, i.e., a lower mean error $\eta$ and an improved overall correlation *R*, relative to the literature constant sets (see Table 8). Also, FT-L has a correlation *R*, which is similar to the best correlation from the literature constants (GW and ZT; see table 8). Nonetheless, it performs generally better than the GW or ZT constants, since the mean error $\eta$ to the experimental hemolysis is significantly reduced compared to the results of GW or ZT. Both, the closer agreement with experimental data as well as the higher correlation of FT-M and FT-L are important factors for design, optimization and comparison of medical devices, since the quantitative value as well as correct trend in hemolysis should be reflected by an applied numerical hemolysis model.

Nonetheless, even the best constant sets from the MOPSO do not perfectly match all 12 operation points with a fitness function of $F_n = 0$. Several reasons on experimental as well as numerical side could be responsible for this mismatch. For example, the experimental hemolysis data used for optimization were obtained from multiple sources that use different donor species. Ding et al. demonstrated that the hemolytical response of porcine and bovine blood, which are used in the present test cases, is similar and also Herbertson et al. showed that the absolute differences in ${\rm MIH_{exp}}$ were small for tests with porcine and bovine blood in the FDA Nozzle (Ding et al. 2015; Herbertson et al. 2015). However, Herbertson’s results also indicate that the relative ratio of hemolysis generated at the nozzle’s OPs is different for the two species, which might impact the optimization.

Another experimental influence on the numerical optimization is the high uncertainty in the hemolysis experiments. We tried to account for this by suggesting that an optimal numerical hemolysis prediction lies within these uncertainties. A smaller uncertainty in the measured data would mean a higher probability of finding a more universal constant set. On the other hand, a higher uncertainty of the experiments allows the optimization more room to hit the experimental results (and reach $F_n=0$). But, even this matching of the optimized results within the high uncertainties was not possible with the performed optimizations. For this reason, we suspect that there is also room for improvement on the numerical side (in model building) so that a quantatively correct hemolysis prediction becomes possible.

Regarding that, a very interesting numerical observation is the fact that it seems to be impossible to optimize the hemolysis prediction in both the capillary tube cases as well as the FDA nozzle cases. The tube cases seem to have surprisingly high ${\rm MIH_{exp}}$ relative to the simulated stress field (the stresses are in the same order of magnitude for both cases, not shown in this paper). It suggests - assuming that the simulated flow fields as well as the experimental hemolysis data are accurate - that incorporating solely the whole stress field in the hemolysis prediction might be inappropriate to properly predict hemolysis. A plausible explanation for this can be found in the single-phase assumption of the flow simulations. It assumes that hemolysis is a global phenomenon that can happen anywhere in the flow domain, while in reality it happens only where erythrocytes are present. When considering blood as a multi-phase flow, literature studies suggest that the erythrocytes experience migratory forces and thus accumulate toward certain areas within the flow domain (Fraser et al. 2012; Stergiou et al. 2019). The stresses in these areas may weigh heavier toward the hemolysis prediction than those where only few erythrocytes can be found. A future hemolysis model that accounts for these multiphase effects could be a milestone for correct a hemolysis prediction.

Another point that should be addressed is the inclusion of turbulence in our hemolysis prediction. Some of the OPs (especially the capillary tube cases) are in a transitional flow regime, which does not automatically mean that a fully turbulent flow is present. Nevertheless, significant turbulent stresses affecting the total stresses (Eq. (10)) are present even at these (and all other) operating points (a figure showing the turbulent and total stresses in the capillary tube at the smallest Reynolds number is given in the Supplementary file 2). Although the contribution of this turbulence is included in our numerical hemolysis prediction, the simulation does not account for the potentially complex interaction of erythrocytes with turbulent eddies (Antiga and Steinman 2009; Morshed et al. 2017), which could affect the “real-acting” stresses, and thus hemolysis in the real blood flow. A fluid model or hemolysis model, which account for this interaction could improve numerical hemolysis prediction, but does not exist to the author’s knowledge.

Since global optimization of the constants was not possible even with a data set of millions of optimized hemolysis results, the question arises whether a generalization of hemolysis prediction with the stress-based model is possible at all. This issue has also been discussed, for example, by Tobin and Manning (2020), with a number of possible reasons. The results of the present study cannot yet answer this question conclusively, but could rather be understood as an indication of the invalidity of a generalizable stress-based model (in the form it is implemented in this study). We believe that two main issues must be solved for the optimization with the MOPSO (or any other optimization algorithm) in order to derive a hemolysis model, which correctly predicts hemolysis after all:

- The implemented stress-based model just correlates variables of the simulated, single-phase flow directly with the amount of hemoglobin released. Hence, it does not take the physical properties of erythrocytes, the mechanisms leading to membrane failure (Faghih and Sharp 2019) or the interaction between the turbulence and the erythrocytes (Antiga and Steinman 2009) into account. For this reason, it might be necessary to use more advanced hemolysis models, such as the aforementioned strain-based models, in order to predict hemolysis in turbulent flows correctly. The optimization algorithm described in this study is capable of handling such models, and will help us in further model developments. Regarding that, the next steps for the authors will be the coupling of more advanced hemolysis models with the MOPSO algorithm to evaluate whether a “better” hemolysis prediction is possible with more advanced models.
- Furthermore, more turbulent experimental test cases are needed for the derivation and optimization of hemolysis models. Thereby, hemolysis should preferably be measured with the same donor species and according to the same protocol. In addition, all important blood and flow parameters (*Hb*, $H_{\rm ct}$, $\Delta fHb$, *Q*, $\Delta t$, ...), which are needed for the simulation validation and hemolysis prediction, should be reported. To the authors’ knowledge, the test cases presented in this paper are one of the few well-documented and freely available turbulent test cases including hemolysis measurements. A larger database with a large number of well-documented test cases would be of immense value not only for the future model development by the authors.

In conclusion, the presented optimization algorithm serves as a foundation with the potential to evolve into a mighty development tool for future hemolysis models. It allows the prediction of numerical hemolysis in only a fraction of the time of a flow simulation and utilizes the swarm optimization to not only find improved constant sets, but also find relations between the constants. The validity of the results can be improved by feeding the algorithm with more experimental data and refining the optimization criteria.

## Supplementary Information

Below is the link to the electronic supplementary material.

Supplementary file 1 (pdf 187 KB)

Supplementary file 2 (pdf 201 KB)
