# Supplementary material for: Flow simulation-based particle swarm optimization for developing improved hemolysis models
Source: Biomech Model Mechanobiol. 2022 Nov 28;22(2):401–16. doi: 10.1007/s10237-022-01653-7 (PMC10097800; doi:10.1007/s10237-022-01653-7)
Supplement: Supplementary file 1 — (pdf 187 KB) [file 10237_2022_1653_MOESM1_ESM.pdf]

**Article**: Flow Simulation-based Particle Swarm Optimization for Developing Improved Hemolysis Models

**Journal**: Biomechanics and Modeling in Mechanobiology

**Authors**: B. Torner*; D. Frank; S. Grundmann; F.-H. Wurm

**Affiliation**: Institute of Turbomachinery, University of Rostock

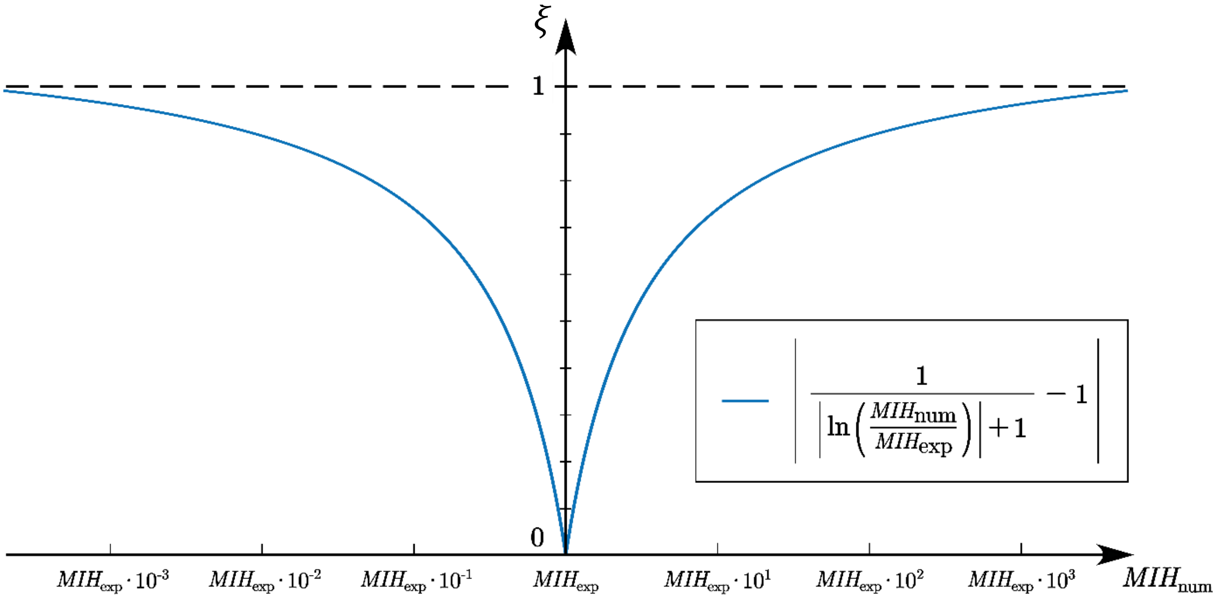


*Supplementary Material A: Visualization of equation 18, which is essentially a modified relative error estimation. The goal of the equation is to equally rank an underestimation and overestimation of the experimental value, while ensuring that the maximum possible error is equal to 1. This is to avoid bias towards small MIH values, as might be the case with RMS and other similar error estimates.*

*benjamin.torner@uni-rostock.de
