# Supplementary material for: Flow simulation-based particle swarm optimization for developing improved hemolysis models
Source: Biomech Model Mechanobiol. 2022 Nov 28;22(2):401–16. doi: 10.1007/s10237-022-01653-7 (PMC10097800; doi:10.1007/s10237-022-01653-7)
Supplement: Supplementary file 2 — (pdf 201 KB) [file 10237_2022_1653_MOESM2_ESM.pdf]

Article: Flow Simulation-based Particle Swarm Optimization for Developing Improved Hemolysis Models

Journal: Biomechanics and Modeling in Mechanobiology

Authors: B. Torner*; D. Frank; S. Grundmann; F.-H. Wurm

Affiliation: Institute of Turbomachinery, University of Rostock

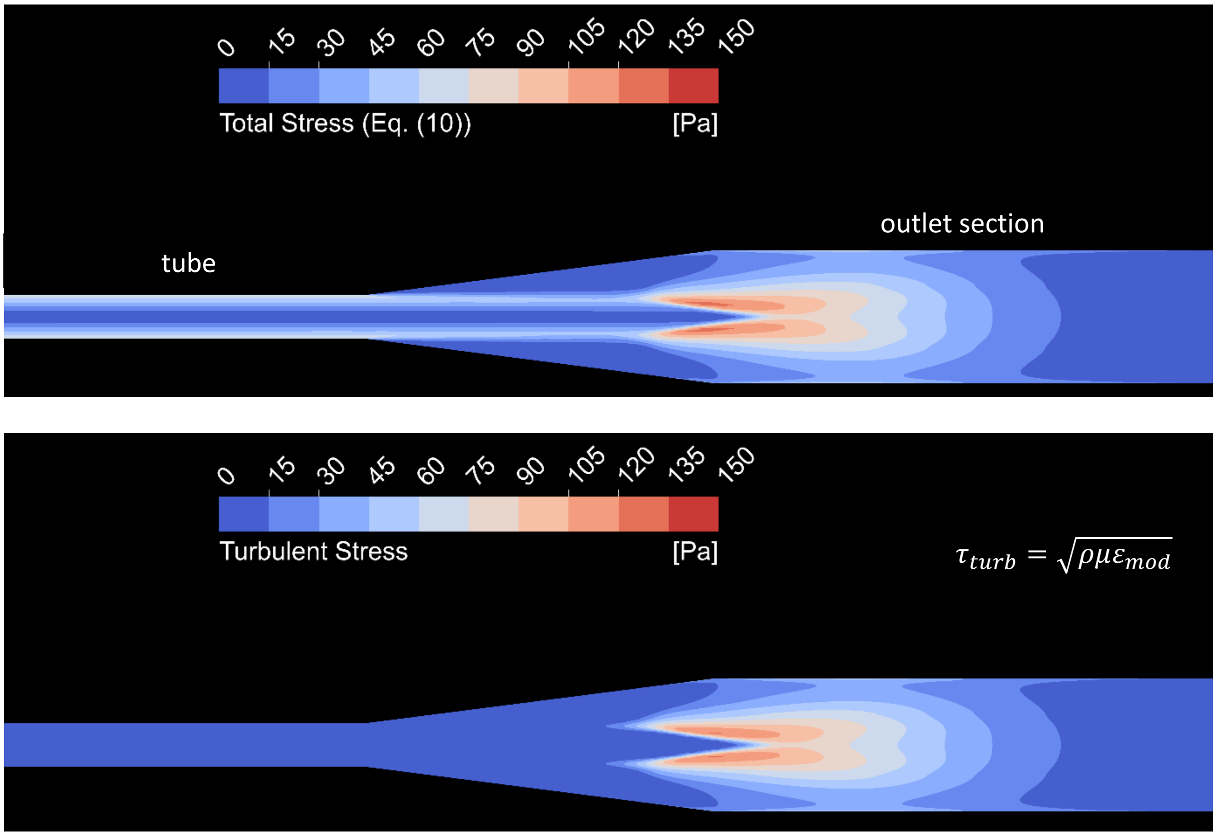

Supplementary Material B: Total and turbulent stresses in a cut-plane through the capillary tube at $Re = 2230$.

*benjamin.torner@uni-rostock.de
